# Successes and Challenges in an Integrated Tuberculosis/HIV Clinic in a Rural, Resource-Limited Setting: Experiences from Kericho, Kenya

**DOI:** 10.1155/2012/238012

**Published:** 2012-02-16

**Authors:** Douglas N. Shaffer, Eunice T. Obiero, Josphat B. Bett, Ignatius N. Kiptoo, Jonah K. Maswai, Fredrick K. Sawe, E. Jane Carter

**Affiliations:** ^1^Kenya Medical Research Institute/Walter Reed Project HIV Program, P.O. Box 1357, Kericho 20200, Kenya; ^2^The Kericho District Hospital, Kenya Ministry of Health, P.O. Box 11, Kericho 20200, Kenya; ^3^Miriam Hospital, Alpert Medical School, Brown University, 164 Summit Avenue, Providence, RI 02908, USA

## Abstract

*Objective*. To describe TB/HIV clinic outcomes in a rural, Ministry of Health hospital. *Design*. Retrospective, secondary analyses. Descriptive statistics and logistic regression analyses evaluated baseline characteristics and outcomes. *Results*. Of 1,911 patients, 89.8% were adults aged 32.0 (±12.6) years with baseline CD4 = 243.3 (±271.0), 18.2% < 50 cells/mm^3^. Pulmonary (84.8%, (32.2% smear positive)) exceeded extrapulmonary TB (15.2%). Over 5 years, treatment success rose from 40.0% to 74.6%, lost to follow-up dropped from 36.0% to 12.5%, and deaths fell from 20.0% to 5.4%. For patients starting ART after TB treatment, those with CD4 ≥ 50 cells/mm^3^ were twice as likely to achieve treatment success (OR = 2.0, 95% CI = 1.3–3.1) compared to those with CD4 < 50 cells/mm^3^. Patients initiating ART at/after 2 months were twice as likely to achieve treatment success (OR = 2.0, 95% CI = 1.3–3.3). Yearly, odds of treatment success improved by 20% (OR = 1.2, 95% CI = 1.0–1.5). *Conclusions*. An integrated TB/HIV clinic with acceptable outcomes is a feasible goal in resource-limited settings.

## 1. Introduction

Tuberculosis (TB) remains the leading cause of death among persons living with HIV/AIDS with nearly 25% of HIV mortality attributable to TB and 380,000 deaths in 2009 related to HIV-associated TB [1]. In 2009, there were an estimated 1.1 million HIV-positive TB patients globally with nearly 80% living in sub-Saharan Africa [1]. While guidelines [2–5] and peer-reviewed journals [6–12] call for integrated TB/HIV services and clinical trials demonstrate early antiretroviral therapy (ART) in patients with TB/HIV coinfection result in better outcomes [13–16], experience from field implementation of TB/HIV integration is now critical. 

 The Kericho District Hospital (KDH), a Ministry of Health public hospital in rural Kenya, fully integrated HIV services into their TB clinic in 2005 quickly following the development of their HIV clinic services. Integration was deemed necessary for a variety of reasons: (1) treatment of one patient with two diseases (TB/HIV) seemed more practical in a single location; (2) both patients and clinicians preferred a combined TB/HIV care delivery system [10]; (3) the belief that treatment outcomes could be maximized by developing a cadre of health care providers capable of providing both TB and HIV treatments; (4) planned increases in HIV testing within the TB clinic would result in identifying large numbers of HIV-infected persons that could overwhelm a burdened HIV clinic (who would already received treatment in the TB clinic); (5) rudimentary infection control in the HIV clinic could be strengthened by caring for all TB/HIV suspects in the TB clinic rather than comingling in the HIV clinic. 

The objectives of this study were to describe characteristics of patients enrolled in the TB/HIV clinic, consider changes in treatment success over time, evaluate TB treatment outcomes given baseline CD4+ T-cell counts and timing of ART, and explore the relationship between baseline characteristics and treatment success.

## 2. Materials and Methods

### 2.1. Study Design

 We conducted a retrospective, secondary analysis of KDH clinic electronic medical records.

### 2.2. Study Setting

 KDH is located in Kericho among the tea fields and plantations of Kenya's southern Rift Valley Province 260 kilometers northwest of Nairobi. As a Ministry of Health (MoH) facility under the Ministry of Medical Services, KDH provides services to a rural, largely uninsured population, representative of the national statistic indicating that 46% of the population lives below the poverty line [17]. In 2010, approximately 12,000 patients received inpatient and approximately 160,000 patients received outpatient services [18]. Both HIV and TB services are provided by funding from the Government of Kenya/MoH [19, 20] and through donor programs, principally the President's Emergency Plan for AIDS Relief (PEPFAR) [21]. TB and HIV care services including HIV Counseling and Testing and Prevention of Mother- to- Child Transmission (PMTCT) operate in partnership with the Kenya Medical Research Institute/Walter Reed Project (KEMRI/WRP) HIV Program. The KEMRI/WRP partnership is part of the US Military HIV Research Program (MHRP), an international HIV vaccine research program under the Walter Reed Army Institute of Research (WRAIR) [22, 23]. 

**Figure 1:**
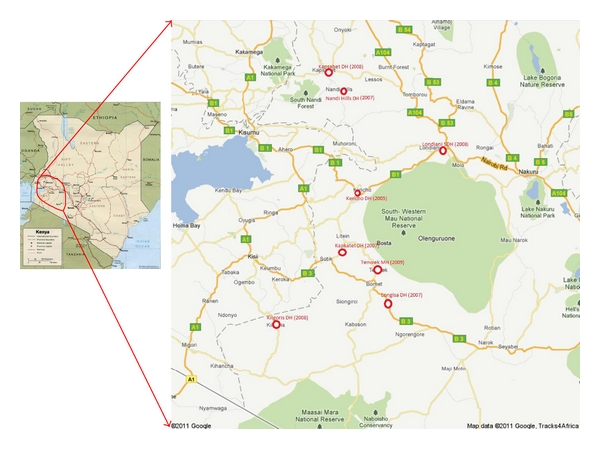
Kericho District Hospital Integrated TB/HIV Clinic (est. 2005) and subsequent roll-out of seven regional integrated TB/HIV clinics (est. 2007–2009). Notes: DH: District Hospital, MH: Mission/Faith Based Hospital, SDH: Sub-District Hospital.

 The MoH in collaboration with KEMRI/WRP under the PEPFAR program supports HIV care, prevention, and treatment services in the southern Rift Valley Province of Kenya (population of approximately 2.5 million). Beginning with 4 initial treatment sites (including KDH) in 2004, program coverage now includes 11 district level, primary treatment facilities, 10 subdistrict hospitals, 75 dispensaries/rural health centers, and 403 PMTCT sites. Expansion has been based upon a network model focusing upon decentralization in an effort to bring services closer to communities and decongest primary treatment centers. After the initial KDH TB/HIV clinic opened in 2005, expansion of integrated TB/HIV services began in 2007 and currently includes 8 TB/HIV integrated clinics (Figure 1).

 In late 2005, KDH opened the integrated TB/HIV clinic using current MoH TB and HIV guidelines as well as expert consultation (EJC). Operational components of the integrated TB/HIV clinic included (1) HIV Diagnostic Testing and Counseling (DTC) for patients and family members presenting to the TB clinic where >90% annual patient acceptance rate was achieved [18]; (2) use of “cough monitors” (trained lay individuals) to maximize sputum collection in an effort to improve case finding and categorization of TB disease, (3) referral to the TB clinic of all patients diagnosed with TB in the HIV clinic at KDH as well as local subdistrict hospitals and dispensaries/rural health centers; and, (4) treatment for HIV including ART in the TB clinic with patient referral for continued care to the HIV clinic on completion of TB therapy. 

 Trimethoprim/sulfamethoxazole prophylaxis is routinely provided to all HIV-infected patients. Patients receive multivitamins for nutritional supplementation as well as pyridoxine while on isoniazid for prevention of peripheral neuropathy. TB, HIV, and TB/HIV coinfection prevention, diagnoses, and treatments evolve based upon current national guidelines and are conducted with support from and under the auspices of Division of Leprosy, Tuberculosis, and Lung Diseases (DLTLD) and National AIDS and STI Control Programme (NASCOP) [3, 24–29]. The TB/HIV clinic is staffed primarily by clinical officers and nurses and is supplemented by DTC staff, cough monitors, and a nutritionist. Medical officers and consultants (mostly Internists) see patients 1-2 days a week or as needed. 

 TB intensive phase treatment consists of isoniazid, rifampin, pyrazinamide, and ethambutol for 2 months in a fixed dose combination with followup weekly. TB continuation phase treatment initially consisted of isoniazid and ethambutol for 6 months in a fixed dose combination until 2009 when country guidelines changed the regimen to isoniazid and rifampin for 4 months in a fixed dose combination. Continuation phase visits were scheduled monthly. First-line ART prescribed in the TB clinic was in accordance with national guidelines and consisted initially of stavudine or zidovudine, lamivudine, and efavirenz until 2010 when tenofovir became available and stavudine was removed from first-line recommendations. TB and ART medications were dispensed on the same schedule. No drug stockouts of TB medications or ART were experienced during the study period. However, postelection violence occurred in Kenya beginning December 29, 2007 and resulted in the displacement of up to 600,000 individuals and over 1,000 deaths. The region of violence included Kericho and resulted in displacement of both patients and staff during the study period.

### 2.3. Data Collection and Variables

 Data were collected from the KDH TB/HIV and HIV clinic electronic medical records systems. These KDH MS Access databases include clinical information from both clinic encounter forms and laboratory results. Data were extracted from both the TB/HIV and HIV clinic databases and merged by patient clinic number to allow evaluation of KDH HIV clinic followup. A final password protected, anonymous database was created for all retrospective, secondary analyses and stored in the KEMRI/WRP Clinical Research Center secured, double-locked Information Department. Basic demographic and medical information extracted included age, gender, baseline CD4+ T-cell count, TB type, treatment and referral categories, and TB treatment outcomes. 

### 2.4. Statistical Analysis

 Descriptive analyses including paired *t*-tests and chi-square or Fisher's exact tests where appropriate were used for baseline characteristics and follow-up outcomes. Unadjusted and adjusted multivariable logistic regression analyses were used to examine the relationship between baseline characteristics, clinic year, and outcomes. Data compilation and statistical analyses were completed using Microsoft Excel and PC SAS (SAS Institute, Carey, NC). Further details are outlined in protocol RV215 [30].

### 2.5. Ethics Approval

 This study was reviewed and approved by the Kenya Medical Research Institute, Lifespan, and Walter Reed Army Institute of Research respective Institutional Review Boards and/or Ethics Review Committees.

## 3. Results

### 3.1. Baseline Characteristics

 Between 2005 and 2010, 1,911 TB/HIV coinfected patients were registered in the TB/HIV clinic: 1,716 adult (52.6% female) and 195 pediatric (58.5% female) (Table 1). Of the adults, 42.8% were in care in the HIV Clinic at the time of their TB diagnosis, resulting in enrollment at the combined clinic. Most adults (51.2%) had their TB diagnosis at the TB clinic and were diagnosed with HIV coinfection by HIV rapid testing in the TB clinic. The remaining adults were referred to the integrated clinic from outside facilities. For pediatric patients, this ratio was reversed with 59.0% cared for in the HIV clinic prior to TB diagnosis and 36.9% having TB diagnosed initially followed by HIV testing in the TB clinic. 

 Most adults (84.8%) presented with pulmonary TB (34.9% smear positive) and advanced HIV disease (mean CD4+ T-cell count = 216.4 ± 206.8 cells/mm^3^, CD4+ T-cell count <50 cells/mm^3^ = 18.4%). Although pediatric patients presented with similar frequencies of pulmonary TB, far fewer were smear positive (8.7%) but their HIV disease was advanced (CD4+ T-cell count <50 cells/mm^3^ = 16.2%). Nearly all received chest X-rays (93.0% and 95.9%, resp.), contributing to diagnosis in smear negative cases.

 Among the 1,911 enrolled in the TB/HIV clinic, 684 (35.8%) initiated ART through care in the integrated clinic while receiving TB therapy. Few patients (186 (9.7%)) were already treated with ART at the time of TB diagnosis and had care transferred to the integrated clinic. Most patients (1,041 (54.5%)) with higher CD4+ T-cell counts received ART in the HIV clinic after completion of TB therapy, reflecting treatment approaches prior to more recent guidelines.

### 3.2. TB Treatment Outcomes

**Figure 2:**
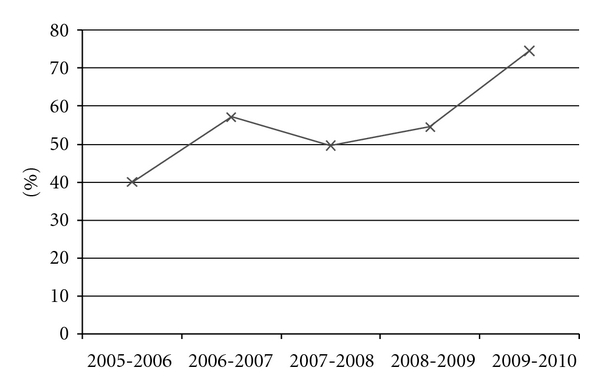
Overall treatment success by year in the Kericho District Hospital TB/HIV Clinic. Note: treatment success defined as proportion with cure or treatment complete.

 Overall 5-year treatment success (cure or completion of therapy) frequencies were 74.7% in children but only 56.8% in adults (Table 2). Proportions lost to followup were high: 14.1% in children and 20.7% in adults. Frequencies of death (5.9% and 8.2%, resp.) were low, although these likely reflect an underestimate if the much larger proportions of lost to followup (14.1% in pediatrics and 20.7% in adults) included patients who died. For adult and pediatric patients over the 5-year period, treatment success rose from 40.0% in 2005 to 74.6% in 2010 (Figure 2). A notable, temporary decrease in treatment success occurred in 2007-2008. This reversal may reflect the societal unrest during the postelection violence that resulted in displacement of both patients and staff. Also during the 5-year period, proportions lost to followup (36.0% year-1 to 12.5% year-5) and having died (20.0% year-1 to 5.4% year-5) also improved.

 In the 684 patients who started ART while on TB therapy, ART was initiated on average 74.0 (±93.1) days following TB treatment initiation. Review of TB treatment outcomes stratified by baseline CD4+ T-cell count distribution reveals that those patients with a CD4 count of <50 cells/mm^3^ had the highest proportion of death (10.7%) and accounted for over half of the deaths in the entire cohort (Table 3(a)). Treatment success frequencies improved in relationship to increasing baseline CD4+ T-cell count. Lost-to-followup frequencies were high (10.9–12.8%) in all CD4+ T-cell strata less than 200 cells/mm^3^. Lost-to-followup frequencies dropped by half in patients with CD4+ T-cell counts above 200 cells/mm^3^. Similarly in review of outcome data by time of ART initiation, poor outcomes as defined by either death or lost to followup were highest in those who started ART earliest following TB treatment initiation, likely reflecting their advanced HIV disease (Table 3(b)). For patients who survived the first two months of TB treatment to initiate ART, treatment success rose to 81.7–92.5%, clearly above the WHO target guidelines.

### 3.3. Baseline Characteristics, Clinic Year, and Treatment Success

 Exploratory analyses were conducted in an effort to understand the relationship between baseline characteristics (age, gender, CD4+ T-cell count, and time until ART initiation), clinic year, and treatment success (cure or treatment complete versus death or loss to followup) (Table 4). Individually, CD4+ T-cell count, time until ART initiation, and clinic year were predictive of treatment success. TB/HIV coinfected patients with CD4+ T-cell counts greater than or equal to 50 or 200 cells/mm^3^ were 2.0 to 3.5 times more likely to achieve treatment success (OR = 2.0 95% CI = 1.3–3.1 and OR = 3.5, 95% CI 1.4–8.9, resp.) compared to patients whose CD4+ T-cell counts were less than 50 or 200 cells/mm^3^. Similarly, patients initiating ART after 2 weeks or 2 months were twice as likely to achieve treatment success (OR = 1.9, 95% CI = 1.0–3.5 and OR = 2.0, 95% CI = 1.3–3.3, resp.). Yearly, the odds of participants having treatment success improved by approximately 20% (OR = 1.2, 95% CI = 1.0–1.5). In multivariate analysis, CD4 category, time until ART, and clinic year remained significant.

## 4. Discussion

 Our experience demonstrates that integrated TB/HIV care is feasible at a rural, public MoH hospital in Kenya. Our integrated clinic, staffed primarily by clinical officers and nurses with backup assistance by medical officers and consultants, successfully administered TB and ART care with acceptable TB treatment success frequencies that improved over time. Overall mortality was low in the TB/HIV clinic for both adult and pediatric patients. However, lost-to-followup frequencies were high and occurred early in care, particularly in those presenting with advanced HIV. Many of these patients lost to followup likely account for additional deaths. Our data suggest that achieving Kenyan national TB targets of 85% cure and 90% treatment completion rates regardless of HIV status can only be achieved if loss to followup is aggressively and promptly addressed. 

 Proportions of patients lost to followup improved from 36% in year 1 to 12.5% in year 5. Interventions employed by the TB/HIV clinic felt to have been beneficial in improving loss to followup rates include (1) early and continuous patient education regarding both TB and HIV therapies by both clinic staff as well as persons living with HIV/AIDS; (2) effort to rapidly identify patients who miss appointments with the use of “tracers” who use village maps obtained with patient permission upon clinic enrollment; (3) use of treatment partners and directly observed therapy (DOT). However, additional qualitative research is required to understand why patients disengage with health care, and innovative solutions are urgently required.

 In parallel with the declining loss to follow-up, treatment success rose and death rates fell over time. One may speculate that these improvements reflect an increased experience and competency in managing well-described complications of TB/HIV treatments including immune reconstitution syndrome, drug interactions, and comorbidities in addition to increased attention to adherence and earlier treatment for patients with lower CD4+ T-cell counts as newer guidelines appeared [4, 5]. Given the Kericho District Hospital served as a regional hospital and was the first to provide comprehensive HIV services in the region, outcomes observed may also reflect a referral bias, which became less pronounced over time as other facilities became available and decentralization of services occurred. 

We found baseline CD4+ T-cell count highly associated with treatment success. Patients with lower CD4 counts most in need of ART were less likely to achieve treatment success compared to those with higher CD4 counts, although treatment success improved over time. These findings underscore challenges in implementing early ART in patients with advanced immunosuppression and achieving results seen in recent clinical trials [14–16]. Challenges of introducing ART early during TB intensive phase treatment in patients with advanced immunosuppression in resource-limited areas are profound, and differences in outcomes observed in clinical trial versus clinical care settings well described in the resource-rich settings are likely magnified in resource-constrained settings [31]. Barriers to early ART are both medical (e.g., pill burden, adherence, toxicities, immune reconstitution syndrome, and comorbidities) and logistical (e.g., frequent appointments requiring travel time and costs, time away from family and work). Clinical trials are better resourced to address these medical and logistical issues, not the case in resource-constrained health care systems. Innovation will be critical in solving these challenges. 

 Hospitalization, while theoretically practical for profoundly immunosuppressed patients who are initiating both TB treatment and ART, may often not be possible or ideal. The lack of strong infection control programs within hospitals in sub-Saharan Africa resulting in nosocomial TB transmission raises a cautionary note [32]. The risk of hospitalization with poor infection control is particularly problematic in areas such as Kenya where TB drug susceptibility data is lacking. Congregation of the most vulnerable (i.e., those with CD4+ T-cell count <50 cells/mm^3^) with those with unknown rates of multidrug or extensively drug resistant TB has led to devastating consequences in South African settings [32]. Hospitalization to overcome challenges and logistical issues of TB/HIV treatments must therefore be initiated with programmatic interventions that can reduce risk. 

“Know your status” campaigns combined with aggressive community-based TB case finding campaigns will offer long-term solutions [4, 5]. The former will avoid discovery of the HIV patient presenting late into care with a low CD4+ T-cell count. The later will reduce transmission risk for all. Until those public health interventions can be broadly implemented, the integrated TB/HIV clinical service must look for solutions to translate clinical trials findings and recommendations into best practice scenarios.

Perhaps the most notable strength of the Kericho integrated TB/HIV clinic was the very early recognition of the need for integrating services (one patient, one clinic) and the institution of an integrated clinic into routine practice at a Ministry of Health facility. The most important limitation to recognize is the retrospective, uncontrolled nature of our analyses and those limitations (e.g., missing data) and biases (e.g., selection, referral, and report biases) inherent in all such secondary analyses. To that end, analyses were identified as exploratory and results are best considered hypotheses supporting or generating. While particular caution should be given to interpretations of pediatric data alone due to the low proportion of pediatric patients, we feel that a strength of the integrated TB/HIV clinic is the inclusion of pediatric patients.

## 5. Conclusions

 TB/HIV patients with advanced HIV disease can be successfully managed in integrated TB/HIV MoH clinics in rural Kenya. Treatment outcomes improve with time, likely reflecting a variety of factors certainly including additional expertise that follows experience. Baseline CD4+ T-cell count is a driving force in predicting outcomes. Loss to followup remains a critical challenge in care delivery that demands attention and innovation. With high loss to followup occurring early in care and likely representing further deaths, the benefits and challenges of early ART initiation in TB treatment in nonclinical trial, resource-limited, rural settings are underscored. This “TB/HIV timeline” conundrum underscores the need for increased efforts for early detection of TB/HIV coinfected patients combined with innovative case holding interventions. An integrated TB/HIV clinic (one patient, one clinic) is a feasible goal in resource-constrained settings.

## Figures and Tables

**Table 1:** Characteristics of TB/HIV coinfected patients upon enrollment in the Kericho District Hospital Integrated TB/HIV Clinic^1^.

	Pediatric (≤15 years old) *n* = 195 (10.2%)	Adult (>15 years old) *n* = 1,716 (89.8%)	Overall *n* = 1,911
Age (yrs)^2^	6.6 (±4.0)	34.9 (±9.7)	32.0 (±12.6)
	6.0 [1–15]	34.0 [16–80]	32.0 [1–80]
Female (%)	58.5	52.6	53.2
CD4+ T-cell count (cells/mm^3^)^2^	551.1 (±568.9)	216.4 (±206.8)	243.3 (±271.0)
	398.5 [2-3,151]	153.0 [1-1,609]	165.0 [1–3, 151]
CD4 distributions (%)^2^			
<50 (cells/mm^3^)	16.2	18.4	18.2
50–99 (cells/mm^3^)	5.4	16.3	15.4
100–199 (cells/mm^3^)	7.7	24.3	22.9
200-349 (cells/mm^3^)	16.2	21.3	20.8
≥350 (cells/mm^3^)	54.6	19.8	22.6
TB type (%)			
(I) Pulmonary	84.1	84.8	84.8
(1.a) Smear positive^2^	8.7	34.9	32.2
(1.b) Chest X-ray done	95.9	93.0	93.3
(II) Extrapulmonary	15.9	15.2	15.2
Treatment category^3^			
New	93.3	89.0	89.5
Transfer in	1.0	3.7	3.4
Returned defaulter	0.5	2.9	2.7
Relapse	5.1	4.4	4.4
Referral origin^2^			
HIV clinic	59.0	42.8	44.5
TB clinic	36.9	51.2	49.8
Private	1.5	3.7	3.5
Other	2.5	2.2	2.2

^1^Data presented as proportion (%) or mean (±SD) and median [range]. Missing data: CD4 = 15.4%; TB type, treatment category, referral, and family testing <1.0%.

^2^ *P* < 0.05.

^3^ *P* < 0.001.

**Table 2:** TB treatment outcomes of TB/HIV coinfected patients enrolled in the Kericho District Hospital Integrated TB/HIV Clinic.

	Pediatric (<15 years old) *n* = 195	Adults (>15 years old) *n* = 1716
TB treatment outcome		
Cure	7 (4.1)	243 (16.0)
Treatment complete	120 (70.6)	621 (40.8)
Transferred out	9 (5.3)	217 (14.3)
Lost to Followup	24 (14.1)	315 (20.7)
Died	10 (5.9)	125 (8.2)
Treatment success	127 (74.7)	864 (56.8)

Notes:

(1) Data presented as number (percentage). Missing data for TB treatment outcome = 12.9% for pediatric and 11.3% for adult patients.

(2) Treatment success defined as cure or treatment complete.

**Table:** (a)

CD4 distributions (cells/mm^3^)	TB treatment outcome				Row totals
	Treatment success	Transferred out	Lost to followup	Died	
<50	116 (66.3)	18 (10.3)	22 (12.8)	19 (10.7)	175 (28.6)
50–99	101 (68.7)	21 (14.3)	16 (10.9)	9 (6.1)	147 (24.0)
100–199	163 (77.3)	18 (8.5)	27 (12.8)	3 (1.4)	211 (34.5)
200–349	53 (86.9)	4 (6.6)	3 (4.9)	1 (3.1)	61 (10.0)
≥350	16 (88.9)	1 (5.6)	1 (5.6)	0 (0)	18 (2.9)

Notes:

(1) Data presented as number (percentage for row total). Data missing: 10.5% for combination of CD4 distributions and TB treatment outcome.

(2) Treatment Success defined as cure or treatment complete.

**Table:** (b)

Time of ART	Tb Treatment Outcome				Row totals
	Treatment success	Transferred out	Lost to followup	Died	
ART Prior to TB Treatment	122 (75.3)	12 (7.4)	14 (8.6)	14 (8.6)	162 (20.5)
ART Following TB Treatment					
<2 weeks	39 (65.0)	5 (8.3)	10 (16.7)	6 (10.0)	60 (7.6)
2 weeks–<2 months	227 (66.8)	50 (14.7)	45 (13.2)	18 (5.3)	340 (43.0)
2–6 months	143 (81.7)	7 (4.0)	15 (8.6)	10 (5.7)	175 (22.2)
>6 months	49 (92.5)	1 (1.6)	2 (3.8)	1 (1.9)	53 (6.7)

Notes:

(1) Data presented as number (percentage of row total). Data missing: 10.1% for combination of time of ART and TB treatment outcome.

(2) Treatment success defined as cure or treatment complete.

**Table 4:** Relationship of treatment success in patients starting antiretroviral therapy after TB treatment in the Kericho District Hospital TB/HIV Clinic (*n* = 684).

	Odds of treatment success (cure or treatment complete) versus death or loss to followup					
Characteristic	Univariate model			Multivariate model^1^		
	OR	95% CI	*P*	OR	95% CI	*P*
Age						
Adult	0.4	0.1–1.5	0.2	—	—	—
Pediatric	reference					
Gender						
Male	0.7	0.5–1.1	0.09	—	—	—
Female	reference					
CD4 Category						
≥50 cells/mm^3^	2.0	1.3–3.1	<0.01	1.7	1.0–2.7	0.04
<50 cells/mm^3^	reference			reference		
CD4 Category						
≥200 cells/mm^3^	3.5	1.4–8.9	<0.01	2.4	0.9–6.3	0.09
<200 cells/mm^3^	reference			reference		
Time Until ART						
≥2 weeks	1.9	1.0–3.5	0.05	1.3	0.6–2.7	0.5
<2 weeks	reference			reference		
Time Until ART						
≥2 months	2.0	1.3–3.3	<0.01	1.8	1.1–3.0	0.03
<2 months	reference			reference		
Clinic Year						
Year (2–5, 1 year increment)	1.2	1.0–1.5	0.02	1.3	1.1–1.6	<0.01
previous year	reference			reference		

Notes:

OR: odds ratio, CI: confidence interval.

^1^Adjusted for CD4 categories, time until ART categories, and clinic year.

## References

[1] WHO TB/HIV Facts 2011. http://www.who.int/tb/challenges/hiv/factsheet_hivtb_2011.pdf.

[2] WHO (2004). Recommendations of the Interim Policy on Collaborative TB/HIV activities. *The Weekly Epidemiological Record*.

[3] National AIDS and STI Control Programme (NASCOP), Ministry of Health (2006). *Guidelines for Implementing Tb-HIV Collaborative Activities in Kenya—What Health Care Workers Need to Know*.

[4] WHO (2010). *Antiretroviral Therapy for HIV Infection in Adults and Adolescents*.

[5] WHO (2011). *Guidelines for intensified tuberculosis case-finding and isoniazid preventive therapy for people living with HIV in resource-constrained settings*.

[6] Mayer KH, Hamilton CD (2010). Synergistic pandemics: confronting the global HIV and tuberculosis epidemics. *Clinical Infectious Diseases*.

[7] Getahun H, Gunneberg C, Granich R, Nunn P (2010). HIV infection-associated tuberculosis: the epidemiology and the response. *Clinical Infectious Diseases*.

[8] Howard AA, El-Sadr WM (2010). Integration of tuberculosis and HIV services in sub-Saharan Africa: lessons learned. *Clinical Infectious Diseases*.

[9] Lönnroth K, Castro KG, Chakaya JM (2010). Tuberculosis control and elimination 2010–50: cure, care, and social development. *The Lancet*.

[10] Harries AD, Zachariah R, Corbett EL (2010). The HIV-associated tuberculosis epidemic-when will we act?. *The Lancet*.

[11] Marais BJ, Raviglione MC, Donald PR (2010). Scale-up of services and research priorities for diagnosis, management, and control of tuberculosis: a call to action. *The Lancet*.

[12] Ghebreyesus TA, Kazatchkine M, Sidibé M, Nakatani H (2010). Tuberculosis and HIV: time for an intensified response. *The Lancet*.

[13] Abdool Karim SS, Naidoo K, Grobler A (2010). Timing of initiation of antiretroviral drugs during tuberculosis therapy. *New England Journal of Medicine*.

[14] Blanc F-X, Sok T, Laureillard D (2011). Earlier versus later start of antiretroviral therapy in HIV-infected adults with tuberculosis. *New England Journal of Medicine*.

[15] Havlir DV, Kendall MA, Ive P (2011). Timing of antiretroviral therapy for HIV-1 infection and tuberculosis. *New England Journal of Medicine*.

[16] Abdool Karim SS, Naidoo K, Grobler A (2011). Integration of antiretroviral therapy with tuberculosis treatment. *New England Journal of Medicine*.

[17] Ministry of State for Planning N.D., and Vision 2030, Kenya (2010). *Poverty Reduction Strategy Paper*.

[18] Kericho District Hospital, Ministry of Health

[19] The Government of Kenya http://www.statehousekenya.go.ke/government/ministries.htm.

[20] Ministry of Health, Government of Kenya http://www.statehousekenya.go.ke/government/health.htm.

[21] The United States President’s Emergency Plan for AIDS Relief. http://www.pepfar.gov/.

[22] The Kenya Medical Research Institute (KEMRI) http://www.kemri.org/.

[23] U.S. Military HIV Research Program (MHRP) http://www.hivresearch.org/home.php.

[24] National AIDS and STI Control Programme (NASCOP), Ministry of Health (2005). *Guidelines for Antiretroviral Drug Therapy in Kenya*.

[25] National AIDS and STI Control Programme (NASCOP), Ministry of Health (2007). *Kenya National Clinical Manual for ART Providers*.

[26] National AIDS and STI Control Programme (NASCOP), Ministry of Health (2008). *National Manual for the Management of HIV-Related Opportunistic Infections and Related Conditions*.

[27] National AIDS and STI Control Programme (NASCOP) http://nascop.or.ke/index.php.

[28] Division of Leprosy, Tuberculosis, and Lung Diseases (2009). *DLTLD Guidelines on management of Leprosy and Tuberculosis*.

[29] Division of Leprosy, Tuberculosis, and Lung Diseases (DLTLD) http://www.nltp.co.ke/.

[30] Shaffer D, Maswai J (2010). Kericho District Hospital Ministry of Health/President’s Emergency Plan for AIDS Relief Combined TB/HIV Clinic: a Retrospective, Anonymous Clinical Record Review of Progress to Date (RV215, WRAIR #1347, KEMRI NRP#017).

[31] Moore RD, Keruly JC, Gebo KA, Lucas GM (2005). An improvement in virologic response to highly active antiretroviral therapy in clinical practice from 1996 through 2002. *Journal of Acquired Immune Deficiency Syndromes*.

[32] Gandhi NR, Moll A, Sturm AW (2006). Extensively drug-resistant tuberculosis as a cause of death in patients co-infected with tuberculosis and HIV in a rural area of South Africa. *Lancet*.

